# Micro 2D-TLC of Selected Plant Extracts in Screening of Their Composition and Antioxidative Properties

**DOI:** 10.1007/s10337-013-2490-y

**Published:** 2013-06-29

**Authors:** Mirosław A. Hawrył, Monika Waksmundzka-Hajnos

**Affiliations:** Department of Inorganic Chemistry, Faculty of Pharmacy, Medical University of Lublin, Chodźki 4A, 20-093 Lublin, Poland

**Keywords:** Micro thin layer chromatography, Two-dimensional thin layer chromatography (2D-TLC), Phenolic compounds, Plant extracts, Antioxidants

## Abstract

Micro two-dimensional separations were performed on polar bonded stationary phases of the type cyanopropyl-silica using non-aqueous eluents (polar modifier dissolved in *n*-heptane) as the first direction eluents and aqueous eluents (organic modifier—MeOH dissolved in water) as the second direction eluents. The chromatographic process was performed in micro scale using 5 × 5 cm plates, small volumes of eluents and 10 μL of plant extracts to obtain satisfying separation. Plates developed in horizontal chambers were dried and observed in UV light (254 nm and 366 m) photographed by digital camera and derivatized by DPPH to detect antioxidants (free radical scavengers) or derivatized by Naturstoff reagent to detect phenolic compounds (characteristic luminescence of some phenolic compounds). The above experiments give the possibility to construct fingerprints for investigated *Polygonum hydropiper*, *Betula verrucosa* and *Pulmonaria officinalis* extracts. It can be used in quality control of the plant material and its antioxidative activity. Novelty of the paper is the micro-scale of the separation by two-dimensional thin layer chromatography mode. For the first time two-dimensional separation of plant extracts on 5 × 5 cm plates in two directions is performed.

## Introduction

The increasing demand of the development of analytical environmental friendly methods contributes to the miniaturization of existing and well-functioning analysis methods. The main advantage of micro methods (HPLC and TLC) is a low consumption of solvents and the fact that the separations are performed on small plates (7 × 7 cm or less). The separation process is realized on the way 5–7 cm, where theoretical plate height is minimal [1, 2]. In this case, the time of analysis is shortened in comparison with classic TLC methods, where the separation is performed on 10–20 cm plates. There are some papers in which the great efficacy of micro-TLC was proved in the analysis of steroids, plant extracts, dyes from *Spirulina maxima* and some fullerenes [3–6].

The instrumentation for micro-TLC is as simple as in HPTLC and TLC. Classic horizontal chambers can be used. Zarzycki et al. [5] constructed the developing chamber for micro-TLC in which the volume of eluent used is 300–1,000 μL depending on its viscosity.

*Polygonum hydropiper L.* also known as smartweed has a long history of herbal use, both in Eastern and Western herbalism. It is not very often used, and it is seen more as a domestic remedy being valued especially for its astringent properties, which makes it useful in treating bleeding, skin problems, diarrhea, etc. The leaves have anti-inflammatory, astringent, carminative, diaphoretic, diuretic, emmenagogue, stimulant, stomachic and styptic properties. They contain rutin, which helps strengthen fragile capillaries and thus helps prevent bleeding. The seeds are carminative, diuretic and stimulant. The whole plant, either on its own or mixed with other herbs, is decocted and used in the treatment of a wide range of ailments including diarrhea, dyspepsia, itching skin, excessive menstrual bleeding and hemorrhoids. A poultice of the plant is used in treating swollen and inflamed areas. In Chinese tests, the plant was ranked 20th in a survey of 250 potential antifertility drugs. A homeopathic remedy is made from the leaves. It is used in the treatment of piles, menstrual pains and other menstrual complaints [7, 8].

The leaves of the herb *Betula verrucosa* Ehrh. (also known as birch) should be dried in the shade at a temperature below 40 °C. Also used for medicinal purposes are flowers, bark, young branches (falls) and tree sap. Among the medicinal compounds that the tree has, there are the flavonoids that give this material diuretic property. Birch is used in cases of urinary tract infections, such as cystitis, calculus, pyelonephritis, among others. In addition, the leaves have in essential oils such compounds as betulinol, which gives febrifuge actions. Birch is also a good antiseptic and healing remedy. The bark of *B. verrucosa* have tannins, which act as astringents. The sap acts as a diuretic and antirheumatic and is used in cases of gout and rheumatism [7].

*Pulmonaria officinalis* L. (also known as lungwort) has a high mucilage content and this makes it useful in the treatment of chest conditions, being of particular benefit in cases of chronic bronchitis. It combines well with other herbs such as coltsfoot (*Tussilago farfara*) in the treatment of chronic coughs including whooping cough and can also be taken to treat asthma. The leaves and flowering shoots are astringent, demulcent, diaphoretic, diuretic, emollient, mildly expectorant and resolvent. They are often used for their healing effect in pulmonary complaints and their mucilaginous nature makes them beneficial in treating sore throats. The leaves can also be used externally to stop bleeding. They are harvested in the spring and dried for later use. A fluid made from the plant is an effective eyewash for tired eyes. A homeopathic remedy is made from the plant. It is used in the treatment of bronchitis, coughs and diarrhea [7].

The main aim of this work is the examination of the possibility of using micro-TLC in two-dimensional separations of some medicinal plant extracts (*P. hydropiper*, *B. verrucosa* and *P. officinalis*) to obtain the information about their composition and antioxidative activity. The antioxidative activity can be measured using 1,1-diphenyl-2-picrylhydrazyl (DPPH) as spraying reagent for post-chromatography derivatization [9]. The success of this investigations can give possibilities to use 5 × 5 cm plates in two-dimensional thin layer chromatography (2D-TLC) experiments for screening of plant extracts for the presence of antioxidants in an easy and inexpensive way.

## Experimental

The plant materials were obtained from the following producers: *Herba Pulmonariae* from Flos (Mokrsko, Poland), *Herba Polygoni hydropiperis* from Herbapol (Kraków, Poland) and *Folium Betulae* from Herbapol (Lublin, Poland).

Five grams of each plant material was weighted out. Each material was closed in the paper case and extracted in Soxhlet apparatus on water bath for 10 h using chloroform to isolate non polar constituents (chlorophylls). Dried plant materials (in the paper case) after the first extraction were once more extracted by methanol by portion of 300 mL during 10 h. The methanolic extract was evaporated to dryness under reduced pressure in the vacuum evaporator. The dry residue was dissolved in methanol solvent and filled with the same solvent in 25 mL flasks. The prepared extracts were filtered using SOR25 0.45 filter and stored in refrigerator.

HPTLC CN F254s 10 × 10 cm (Merck, Darmstadt, Germany) cut to the 5 × 5 cm squares were used in all experiments. Solvents: propan-2-ol, tetrahydrofuran, *n*-heptane and methanol pro analysis grade were purchased from Polish Reagents (POCh, Gliwice, Poland). Distilled water was mixed with methanol to obtain aqueous phases and *n*-heptane was mixed with propan-2-ol, tetrahydrofuran or ethyl methyl ketone to obtain non-aqueous eluents for 2D-HPTLC.

All test substances listed in Table 1 were acquired from various manufacturers (Sigma, Aldrich, Fluka, Roth). 2,2’-Diphenyl-1-picrylhydrazyl (DPPH) was from Aldrich and used as 0.2 % solution in methanol. 2-(Diphenylboryoxy)-ethylamine and PEG4000-Naturstoff reagent was produced by Merck (Darmstadt, Germany) and used as 5 % methanolic solution of PEG and 1 % methanolic solution of 2-(diphenylboryoxy)-ethylamine.

**Table 1:** Substances investigated

	Substance	Manufacturer
1	Naringin	Sigma
2	Hesperidin	Aldrich
3	Naringenin	Aldrich
4	Hesperetin	Aldrich
5	Flavone	Sigma
6	Apigenin 7-glucoside	Roth
7	Quercetin	Sigma
8	Rutin	Sigma
9	Chlorogenic acid	Aldrich
10	Resveratrol	Roth
11	Myricetin	Aldrich
12	Hyperoside	Aldrich
13	Acacetin	Aldrich
14	Ferulic acid	Fluka
15	Gallic acid	Fluka
16	Caffeic acid	Fluka
17	*p*-Coumaric acid	Fluka

The 0.1 % (w/v) solution of the mixture of test substances (10 μL) and 10 μL methanolic solutions of extracts were spotted by Desaga TLC AS30 applicator (Desaga, Heidelberg, Germany) 0.5 cm from each edge of the chromatographic plate and developed in two directions by use of DS-II developing chambers (Chromdes, Lublin, Poland). In the first direction, the plate was developed using non-aqueous solvent and after drying in air the same plate was developed using aqueous eluent in the direction perpendicular to the first one. In case of non-aqueous solutions, each plate was conditioned in eluent vapours during 15 min to avoid the demixing effect; in case of RP systems (aqueous eluents), plates were not conditioned. After drying, the plates were sprayed using Merck TLC sprayer using 2-(diphenylboryoxy)-ethylamine and PEG4000 (Merck, Darmstadt, Germany and photographed in Camag Cabinet UV lamp at 254 and 365 nm) of Fuji 8 mpx camera or sprayed by DPPH and photographed in day light.

Each separation was replicated three times.

## Results and Discussion

**Fig. 1:**
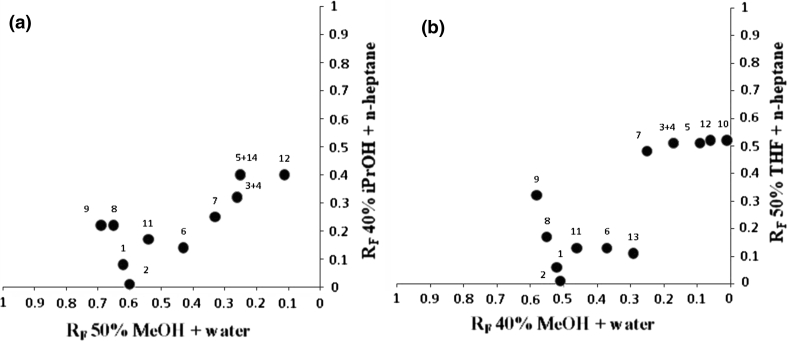
**a** Simulated map of the separation of standards as a plot of R_F_ vs. R_F_ for the systems: I—40 % iPrOH + *n*-Hp; II—50 % MeOH + water. Numbers as in Table 1 **b** Simulated map of the separation of standards as a plot of R_F_ vs. R_F_ for the systems: I—50 % THF + *n*-Hp; II—40 % MeOH + water. Numbers as in Table 1

On the basis of our recent experiments [10], the preliminary choice of optimal eluent systems for the separation of phenolic fraction was performed. Next, the new experiments were carried out to devise chromatographic systems for achieving full separation of standards used in the search. It was stated that optimal NP systems for separation of standards were: 40 % propan-2-ol (iPrOH) in *n*-heptane (*n*-Hp)(v/v) and 50 % tetrahydrofuran (THF) in *n*-heptane (v/v), whereas, optimal RP aqueous eluent was 50 and 40 % methanol (MeOH) in water, respectively. The *R*_*F*_ vs. *R*_*F*_ correlations were made to show the 2D-TLC separation of standards using selected pairs of solvents (Fig. 1a, b). On the basis of these experiments, the two-dimensional separations of standards were performed as well as extracts of investigated plant materials.

Documentation of chromatographic experiments includes pictures of plates photographed in Camag Cabinet UV lamp at 254 and 365 nm after derivatization with the Naturstoff reagent as well as in daylight after spraying with solution of DPPH by use of Fuji 8 mpx camera.

**Fig. 2:**
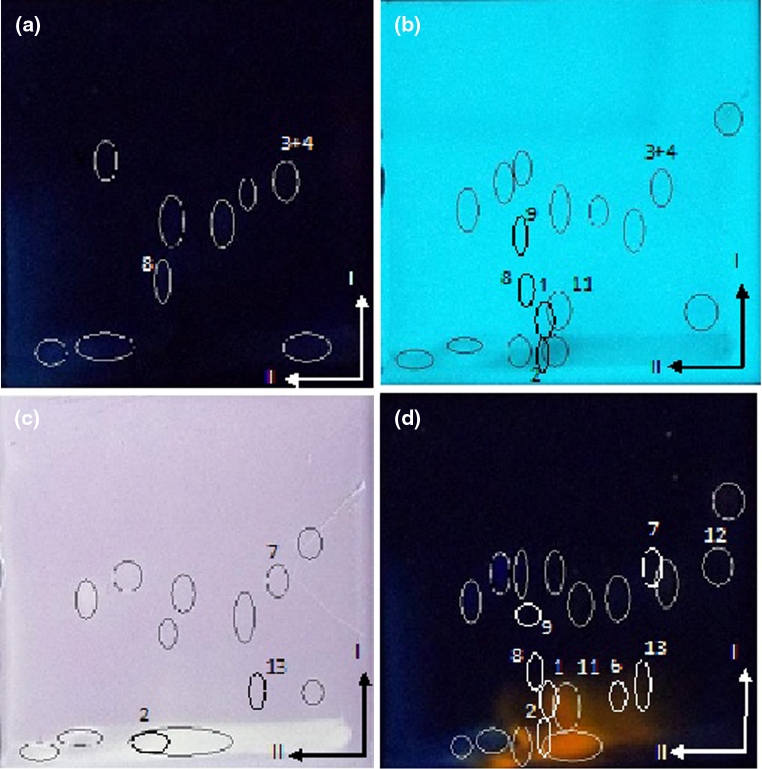
Photographs of chromatographic cyanopropyl plates developed by 2D-TLC mode (I—50 % THF + *n*-Hp; II—40 % MeOH + water) with separated extract of *Polygonum hydropiper*. **a** at λ = 366 nm; **b** at λ = 254 nm **c** after derivatization with DPPH in daylight **d** after derivatization with Naturstoff reagent at λ = 366 nm. Numbers as in Table 1

On the basis of chromatograms of standards (see Table 1), an attempt to investigate some extract components was performed. In the extract of *P. hydropiper* (Fig. 2) developed in system II, the following components were identified: naringin, hesperidin, naringenin, hesperetin, flavone, apigenin 7-glucoside, rutin, chlorogenic acid, myricetin and acacetin.

**Fig. 3:**
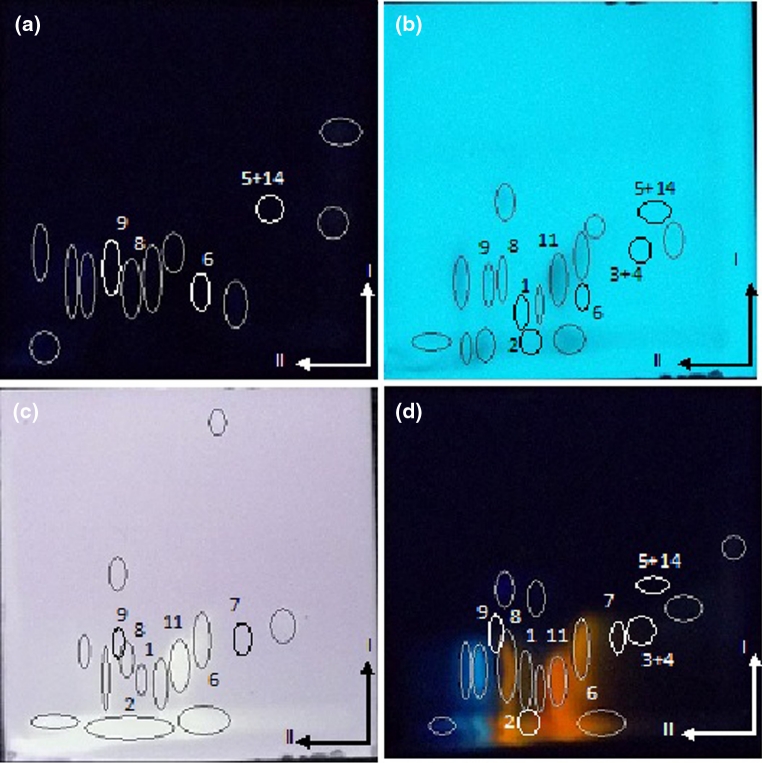
Photographs of chromatographic cyanopropyl plates developed by 2D-TLC mode (I—40 % iPrOH + *n*-Hp; II—50 % MeOH + water) with separated extract of *Betula verrucosa*. **a** at λ = 366 nm; **b** at λ = 254 nm **c** after derivatization with DPPH in daylight **d** after derivatization with Naturstoff reagent at λ = 366 nm. Numbers as in Table 1

The extract of *B. verrucosa* contained naringin, hesperidin, hesperetin, apigenin 7-glucoside, rutin, chlorogenic acid, myricetin, hyperoside, acacetin and gallic acid (Fig. 3).

**Fig. 4:**
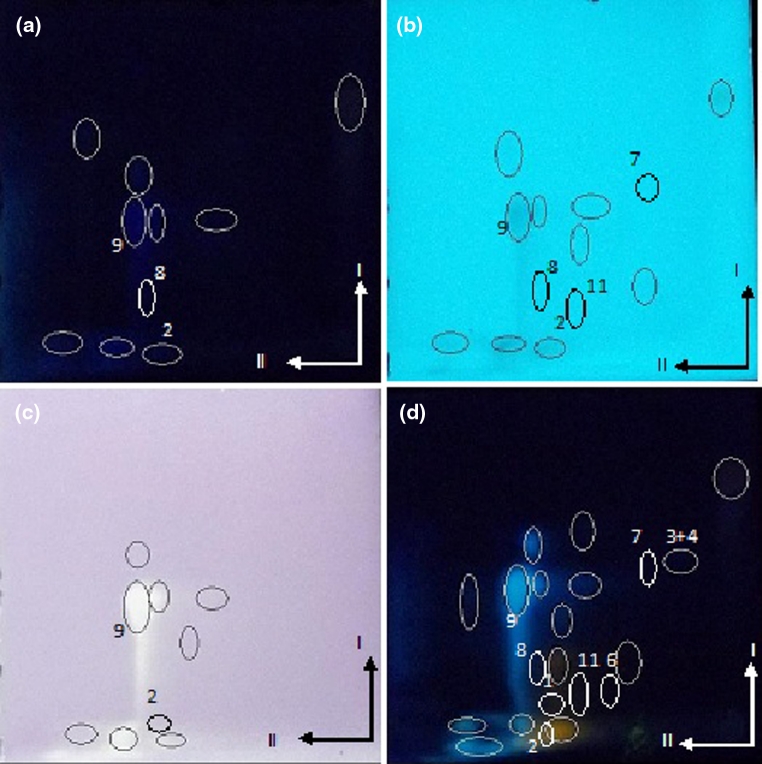
Photographs of chromatographic cyanopropyl plates developed by 2D-TLC mode (I—50 % THF + *n*-Hp; II—40 % MeOH + water) with separated extract of *Pulmonaria officinalis*. **a** at λ = 366 nm; **b** at λ = 254 nm **c** after derivatization with DPPH in daylight **d** after derivatization with Naturstoff reagent at λ = 366 nm. Numbers as in Table 1

The extract from *P. officinalis* contained naringin, hesperidin, apigenin 7-glucoside, rutin, chlorogenic acid, myricetin, hyperoside, acacetin and gallic acid in system I and hesperidin, naringenin, hesperetin, apigenin 7-glucoside, rutin, chlorogenic acid and hyperoside in system II (see Fig. 4).

It should be also emphasized that all micro 2D-TLC chromatograms can be used as fingerprint for each investigated plant material because the differences in extracts’ composition can be also noticed.

The antioxidative activity of fractions of phenolic compounds of investigated extracts can be performed using stable free radical DPPH (purple) giving with free radical scavengers yellow non-radical form. The effect of derivatizing using DPPH in examination of antioxidative activity of extracts is seen in Figs. 2, 3 and 4c. It is clearly seen that only some of the fractions’ components exhibit such activity. There are especially chlorogenic acid (9), 7-Glucoside–Apigenin (6), rutin (8), hyperoside (12), myricetin (11), naringin (1), hesperidin (2), hesperetin (4), gallic acid (15), and quercetin (7).

## Conclusions

Micro-2D-TLC is a useful technique for two-dimensional separation of herbal extracts.

For separation of phenolic fraction of herb material in two-dimensional mode useful is system consisted of cyanopropyl layers developed with non-aqueous eluent composed of propan-2-ol or tetrahydrofuran and *n*-heptane in the first direction and aqueous one composed of methanol and water.

Micro-2D-TLC experiments give the information about composition as well as about antioxidative activity of plant extracts and are helpful in the construction of fingerprints of examined herbs.
